# Supplementary material for: Polyether Ether Ketone Coated with Ultra-Thin Films of Titanium Oxide and Zirconium Oxide Fabricated by DC Magnetron Sputtering for Biomedical Application
Source: Materials (Basel). 2022 Nov 14;15(22):8029. doi: 10.3390/ma15228029 (PMC9694952; doi:10.3390/ma15228029)
Supplement: Supplementary file 1 [file materials-15-08029-s001.zip › materials-1990629-supplementary.pdf]

Support Information of: Polyether ether ketone coated with ultra-thin films of titanium oxide and zirconium oxide fabricated by DC magnetron sputtering for biomedical application

Igor O. Akimchenko[1], Sven Rutkowski[1,2,*], Tuan-Hoang Tran[1], Gleb E. Dubinenko[1], Vsevolod I. Petrov[2], Anna I. Kozelskaya[1], Sergei I. Tverdokhlebov[1,*]

[1] Tomsk Polytechnic University, 30, Lenin Avenue, Tomsk, 634050, Russian Federation
[2] Tomsk Scientific Center of the Siberian Branch of the Russian Academy of Sciences, 10/4, Akademicheskii Prospekt, Tomsk, 634055, Russian Federation
* Correspondence: rutkowski_s@tpu.ru (S.R.), tverd@tpu.ru (S.I.T.)

Supporting Figures:

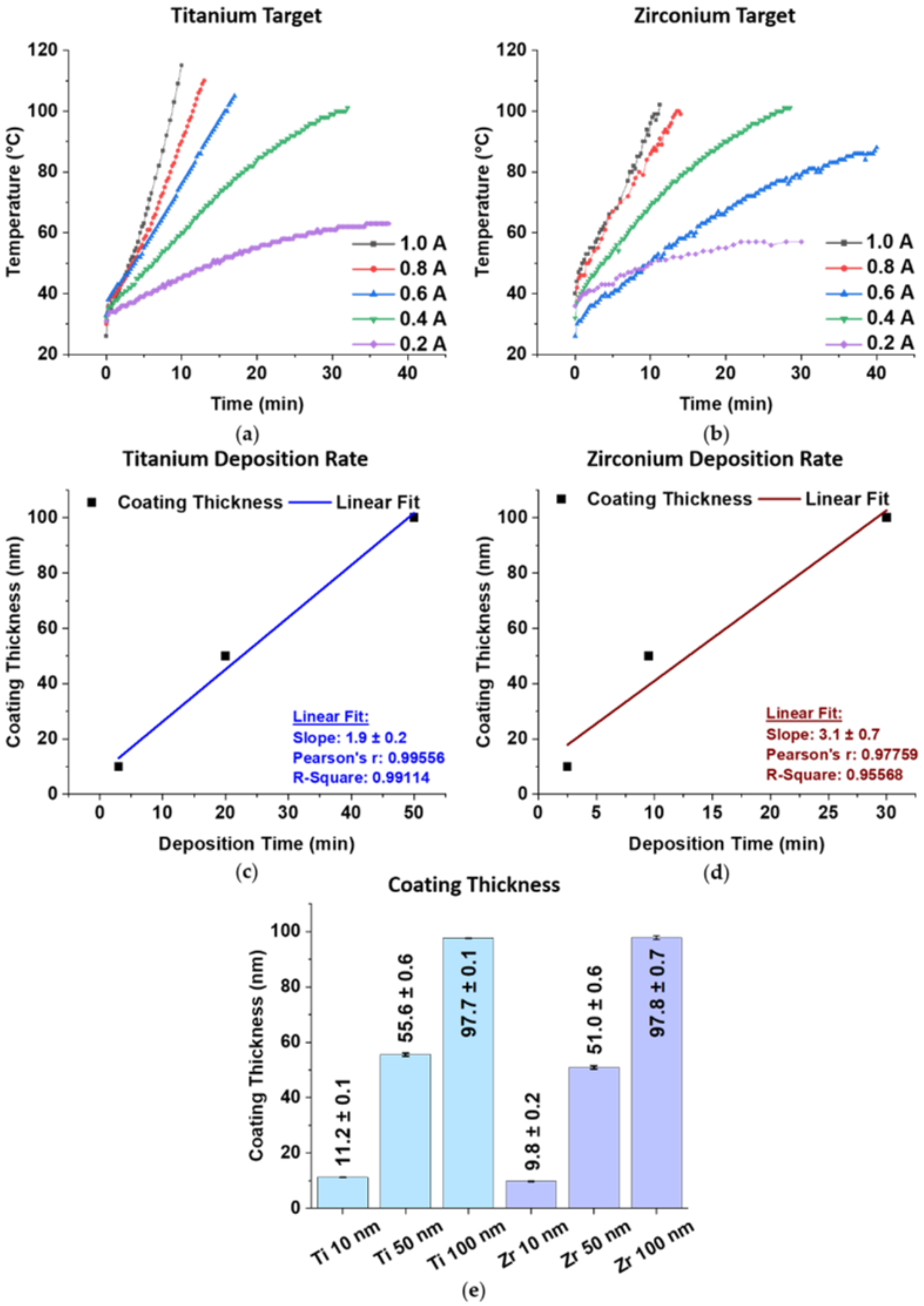

Figure S1. Relationship between sample holder temperature and DC magnetron sputtering time at current values from 0.2 A to 1.0 A for (**a**) Ti target and (**b**) Zr target. Obtained coating thickness for the applied deposition times for (**c**) Ti target and (**d**) Zr target. (**e**) Resulting coating thickness range determined by a spectral ellipsometer using Si wafers for magnetron sputtering.

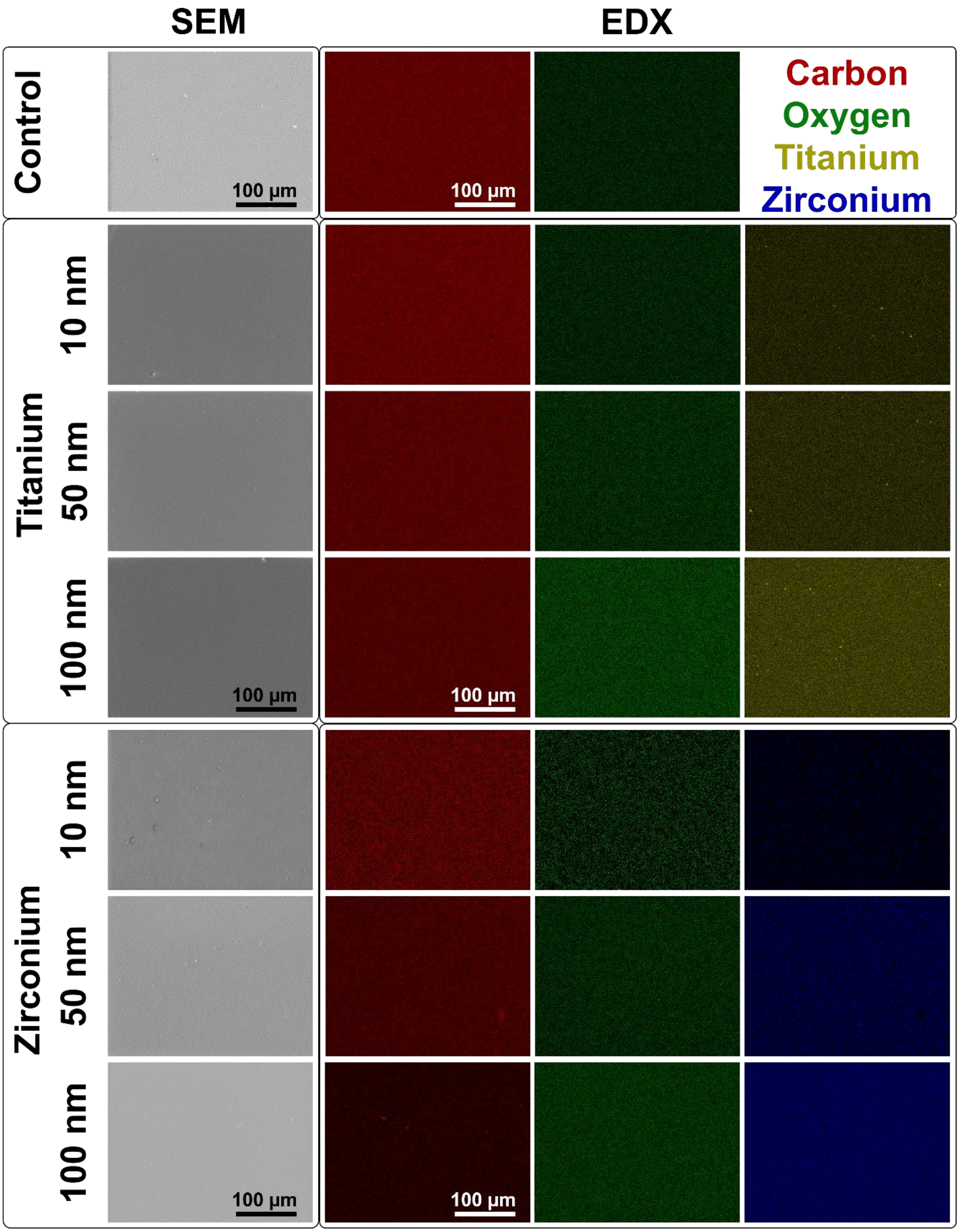

Figure S2. SEM and EDX mapping micrographs of the investigated PEEK sample coatings. Left column SEM micrographs; after that following columns are the EDX micrographs for the following elements: carbon (red), oxygen (green), titanium (yellow) and zirconium (blue/violet). The scale bar is 100 µm for each micrograph.

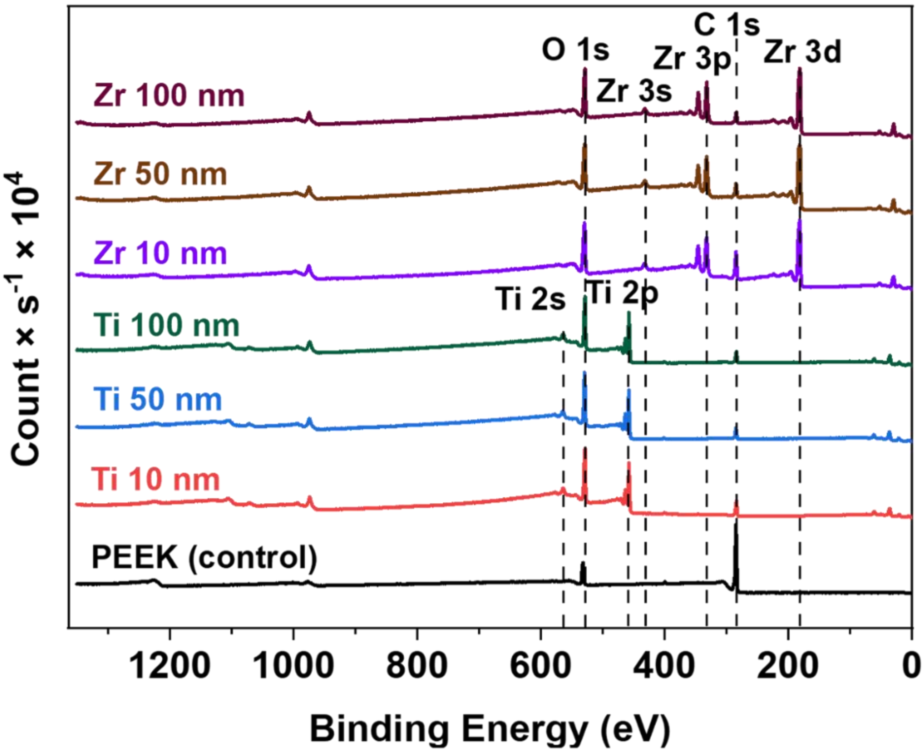

Figure S3. XPS survey spectra of all investigated PEEK samples.
